# Cancer Treatment Using Peptides: Current Therapies and Future Prospects

**DOI:** 10.1155/2012/967347

**Published:** 2012-12-20

**Authors:** Jyothi Thundimadathil

**Affiliations:** American Peptide Company Inc., Sunnyvale, CA 94086, USA

## Abstract

This paper discusses the role of peptides in cancer therapy with special emphasis on peptide drugs which are already approved and those in clinical trials. The potential of peptides in cancer treatment is evident from a variety of different strategies that are available to address the progression of tumor growth and propagation of the disease. Use of peptides that can directly target cancer cells without affecting normal cells (targeted therapy) is evolving as an alternate strategy to conventional chemotherapy. Peptide can be utilized directly as a cytotoxic agent through various mechanisms or can act as a carrier of cytotoxic agents and radioisotopes by specifically targeting cancer cells. Peptide-based hormonal therapy has been extensively studied and utilized for the treatment of breast and prostate cancers. Tremendous amount of clinical data is currently available attesting to the efficiency of peptide-based cancer vaccines. Combination therapy is emerging as an important strategy to achieve synergistic effects in fighting cancer as a single method alone may not be efficient enough to yield positive results. Combining immunotherapy with conventional therapies such as radiation and chemotherapy or combining an anticancer peptide with a nonpeptidic cytotoxic drug is an example of this emerging field.

## 1. Introduction

Mortality from cancer is about to surpass that from cardiovascular diseases in near future. About 7 million people die from cancer-related cases per year, and it is estimated that there will be more than 16 million new cancer cases every year by 2020 [1, 2]. Cancer is characterized by uncontrolled division of cells and the ability of these cells to invade other tissues leading to the formation of tumor mass, vascularization, and metastasis (spread of cancer to other parts of the body) [3]. Though angiogenesis (growth of new blood vessels from preexisting vessels) is a normal and vital process in growth and development, it is also a fundamental step in the transition of tumors from a dormant state to a malignant one [4]. Chemotherapy is one of the major approaches to treat cancer by delivering a cytotoxic agent to the cancer cells. The main problem with the conventional chemotherapy is the inability to deliver the correct amount of drug directly to cancer cells without affecting normal cells [5]. Drug resistance, altered biodistribution, biotransformation, and drug clearance are also common problems [5]. Targeted chemotherapy and drug delivery techniques are emerging as a powerful method to circumvent such problems [6–10]. This will allow the selective and effective localization of drugs at predefined targets (e.g., overexpressed receptors in cancer) while restricting its access to normal cell thus maximizing therapeutic index and reducing toxicity.

 Discovery of several protein/peptide receptors and tumor-related peptides and proteins is expected to create a “new wave” of more effective and selective anticancer drugs in the future, capturing the large share of the cancer therapeutic market [6, 8, 11]. The “biologics” treatment option against cancer includes the use of proteins, monoclonal antibodies, and peptides. The monoclonal antibodies (mAbs) and large protein ligands have two major limitations compared to peptides: poor delivery to tumors due to their large size and dose-limiting toxicity to the liver and bone marrow due to nonspecific uptake into the reticuloendothelial system. The use of such macromolecules has therefore been restricted to either vascular targets present on the luminal side of tumor vessel endothelium or hematological malignancies [12–17]. Peptides possess many advantages, such as small size, ease of synthesis and modification, tumor-penetrating ability, and good biocompatibility [18, 19]. Peptide degradation by proteolysis can be prevented by chemical modifications such as incorporation of D-amino acids or cyclization [18].

Over the years peptides have been evolved as promising therapeutic agents in the treatment of cancer, diabetes, and cardiovascular diseases, and application of peptides in a variety of other therapeutic areas is growing rapidly. Currently there are about 60 approved peptide drugs in the market generating an annual sale of more than $13 billion [18]. Out of four peptide drugs in the market which have reached global sales over $1 billion, three peptides are used in treating cancer directly or in the treatment of episodes associated with certain tumors (leuprolide, goserelin, and octreotide). The number of peptide drugs entering clinical trials is increasing steadily; it was 1.2 per year in the 1970s, 4.6 per year in the 1980s, 9.7 per year in the 1990s, and 16.8 per in 2000s [19]. There are several hundred peptide candidates in the clinic and preclinic development. From 2000 onwards, peptides entering clinical study were most frequently for indications of cancer (18%) and metabolic disorders (17%) [20].

**Figure 1:**
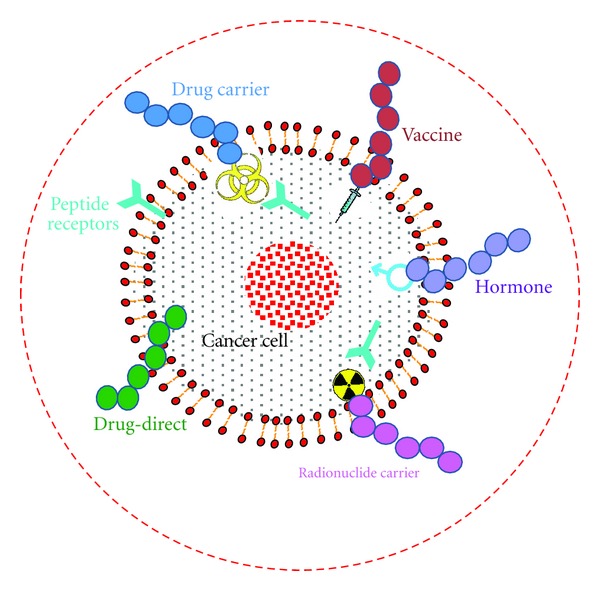
Different possible treatment options of cancer using peptides. Peptides can be used as anticancer drug, cytotoxic drug carrier, vaccine, hormones, and radionuclide carrier.

This paper focuses on different strategies of employing peptides in cancer treatment and management. A special emphasis is given to current peptide drugs available in the market for treating cancer and also peptide candidates in clinical and preclinical stages of development. Peptides can be utilized in a number of different ways in treating cancer [8–10, 19]. This includes using peptides directly as drugs (e.g., as angiogenesis inhibitors), tumor targeting agents that carry cytotoxic drugs and radionuclides (targeted chemotherapy and radiation therapy), hormones, and vaccines. Different possible cancer treatment options using peptides are summarized in Figure 1. Due to the ability to bind to different receptors and also being part of several biochemical pathways, peptides act as potential diagnostic tool and biomarkers in cancer progression. Out of these different possibilities, peptide drugs currently available in the market come from peptide hormone therapy and tumor targeting agents carrying radionuclides (peptide-receptor radio nuclide therapy and imaging). Exceptions to these are two short chain peptide-related drugs, bortezomib and mifamurtide [21, 22]. There is a tremendous progress in other areas such as peptide-vaccine development and peptide angiogenesis inhibitors, and several clinical trials are underway which is expected to bear fruit in near future providing better options to millions of cancer patients. 

## 2. Peptide Hormones: LHRH Agonists and Antagonists

The best classical example of the application of peptides in cancer treatment is the use of LHRH (luteinizing hormone-releasing hormone) agonists introduced by Schally et al. as a therapy for prostate cancer [23–25]. Since then, depot formulations of LHRH agonists such as buserelin, leuprolide, goserelin, and triptorelin have been developed for more efficacious and more convenient treatment of patients with prostate cancer [26–28]. Administration of these peptides causes downregulation of LHRH receptors in the pituitary, leading to an inhibition of follicle-stimulating hormone (FSH) and LH release, and a concomitant decrease in testosterone production. This offered a new method for androgen deprivation therapy in prostate cancer patients. Discovery of LHRH antagonists resulted in therapeutic improvement over agonists as they cause an immediate and dose-related inhibition of LH and FSH by competitive blockade of the LHRH receptors. To date, many potent LHRH antagonists are available for the clinical use in patients. Cetrorelix was the first LHRH antagonist given marketing approval and, thus, became the first LHRH antagonist available clinically [29]. Subsequently new generation LHRH antagonists such as abarelix and degarelix have been approved for human use [30, 31]. A list of LHRH agonists and antagonists available in the market is shown in Table 1.

## 3. Peptide as Radionuclide Carrier: Somatostatin Analogues in Cancer Therapy and Peptide Receptor Radionuclide Therapy (PRRT)

Apart from the use of peptide-based LHRH agonists and antagonists for treating cancer, somatostatin analogues are the only approved cancer therapeutic peptides in the market [32]. Potent analogues of somatostatin (peptide hormone consisting of 14 amino acids, found in *δ* cells of the pancreas as well as in hypothalamic and other gastrointestinal cells) including octreotide (sandostatin) have been developed for the treatment of acromegaly, gigantism, thyrotropinoma, diarrhea and flushing episodes associated with carcinoid syndrome, and diarrhea in patients with vasoactive intestinal peptide-secreting tumors (VIPomas) [33]. Similarly, another long-acting analogue of somatostatin, lanreotide (somatuline), is used in the management of acromegaly and symptoms caused by neuroendocrine tumors, most notably carcinoid syndrome and VIPomas [34].

Most neuroendocrine tumors (NETs) feature a strong overexpression of somatostatin receptors, mainly of subtype 2 (sst2). Currently five somatostatin receptor subtypes (sst) are known (sst1-5) [32, 35]. The density of these receptors is vastly higher than on nontumor tissues. Therefore, somatostatin receptors are attractive targets for delivery of radioactivity via radiolabeled somatostatin analogs. The sst2 has been shown to internalize into the cell in a fast, efficient, and reversible manner after specific binding of a receptor agonist. This molecular process is likely to be responsible for the high and long-lasting uptake of radioactivity in the target cell after binding of the radiolabeled somatostatin analog. Introduced in the late 1980s, [111In-DTPA]-octreotide (Octreoscan), the first available radiolabeled somatostatin analog, rapidly became the gold standard for diagnosis of sst-positive NETs [36, 37]. Numerous peptide-based tracers targeting somatostatin receptors have been developed over the past decade [36, 37]. Octreoscan and NeoTect (tc-99m depreotide) are the only radiopeptide tracers on the market approved by the Food and Drug Administration [37, 38]. An octreotide scan or octreoscan is a type of scintigraphy used to find carcinoid and other types of tumors and to localize sarcoidosis. Octreotide, a drug similar to somatostatin, is radiolabeled with indium-111 and is injected into a vein and travels through the bloodstream. The radioactive octreotide attaches to tumor cells that have receptors for somatostatin. A radiation-measuring device detects the radioactive octreotide and makes pictures showing where the tumor cells are in the body. NeoTect is a radioactive imaging test used to evaluate certain lung lesions in patients who test positive for lung lesions using other imaging tests (e.g., CT or MRI) and have been diagnosed with cancer or have a strong likelihood of cancer. NeoTect identifies certain cells that may be associated with lung cancer and sometimes with other conditions [38].

**Figure 2:**
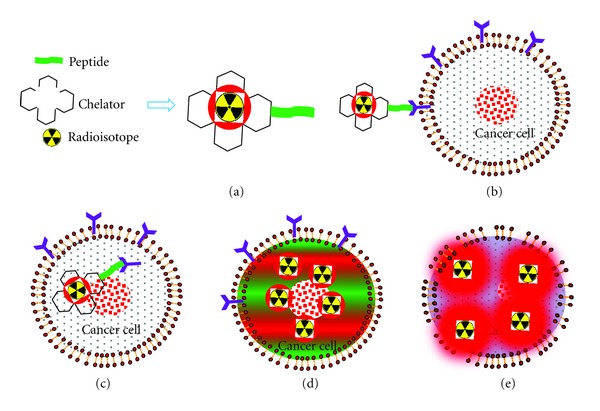
Peptide receptor radionuclide therapy (PRRT); radiolabeled somatostatin analogs generally comprise three main parts: a cyclic octapeptide (e.g., Tyr3-octreotide or Tyr3-octreotate), a chelator (e.g., DTPA or DOTA), and a radioactive element. Radioisotopes commonly used in PRRT are 111In, 90Y, and 177Lu.

Peptide receptor radionuclide therapy (PRRT) combines octreotide (or other somatostatin analogs) with a radionuclide (a radioactive substance) to form highly specialized molecules called radiolabeled somatostatin analogues or radiopeptides [39–48]. Radiolabeled somatostatin analogs generally comprise three main parts: a cyclic octapeptide (e.g., octreotide), a chelator (e.g., DTPA or DOTA), and a radioactive element (111In, 90Y, or 177Lu). These radiopeptides can be injected into a patient and will travel throughout the body binding to carcinoid tumor cells that have receptors for them. Once bound, these radiopeptides emit radiation and kill the tumor cells they are bound to (Figure 2). PRRT using [111In-DTPA]-octreotide (where DTPA is diethylenetriamine pentaacetic acid) is feasible because, besides gamma radiation, 111In emits both therapeutic Auger and internal conversion electrons having tissue penetration ability [39, 40]. However, studies have shown that 111In-coupled peptides are not efficient for PRRT, as the short distance traveled by Auger electrons after emission means that decay of 111In has to occur close to the cell nucleus to be tumoricidal [39, 40]. It was found that replacement of phenylalanine by tyrosine as the third amino acid in the octapeptide leads to an increased affinity for somatostatin-receptor subtype 2. This resulted in the development of next generation therapy using 90Y-DOTA, Tyr3-octreotide [41–44]. This compound has DOTA (tetraazacyclododecane tetraacetic acid) instead of DTPA as the chelator, which allows stable binding of 90Y, a *β*-emitting radionuclide. Various clinical trials around the world showed that it is better than [111In-DTPA]-octreotide in treating gastroenteropancreatic neuroendocrine tumors (GEPNETs). A third generation of somatostatin-receptor-targeted radionuclide therapies was introduced using 177Lu-DOTA, Tyr3-octreotate [49, 50]. The only difference between DOTA, Tyr3-octreotate and DOTA, Tyr3-octreotide is that the C-terminal threoninol of DOTA, Tyr3-octreotide is replaced with the amino acid, threonine. As a result, DOTA, Tyr3-octreotate displays improved binding to somatostatin-receptor-positive tissues when compared with DOTA, Tyr3-octreotide [49]. Gastroenteropancreatic tumors predominantly express subtype 2 of the somatostatin receptor, and DOTA, Tyr3-octreotate has a sixfold to ninefold increased affinity for this receptor subtype *in vitro* compared with DOTA, Tyr3-octreotide [43, 49, 50]. 177Lu-octreotate was very successful in terms of tumor regression and survival in an experimental model in rats. 177Lu-labeled somatostatin analogs have an important practical advantage over their 90Y-labeled counterparts: 177Lu is not a pure *β* emitter, but also emits low-energy *γ* rays, which allows direct posttherapy imaging and dosimetry. Treatment with 177Lu-octreotate resulted in a survival benefit of several years and markedly improved quality of life. PRRT might soon become the therapy of choice for patients with metastatic or inoperable GEPNETs. Nowadays, different somatostatin analogs are available not only for therapeutic purposes but also when labeled with b1-emitters (e.g., 68Ga and 64Cu) for tumor imaging with integrated PET/CT scanners [51, 52]. The PET/CT technology provides a highly valuable combination of physiologic and anatomic information and has been shown to impact significantly on the patient's management. 

Tumor imaging and PRRT have been extended to many other receptors such as Gastrin-releasing peptide/bombesin (GRP) and Cholecystokinin (CCK) in recent years [53, 54]. Radiolabelled receptor antagonists are also emerging as alternatives in this area [55, 56]. 

## 4. Peptide Vaccines

**Figure 3:**
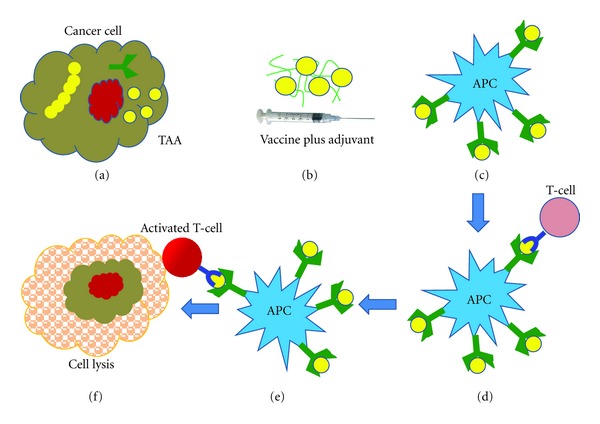
Peptide-based cancer vaccines: tumor cells express antigens known as tumor-associated antigens (TAAs) that can be recognized by the host's immune system (a). These TAAs mixed with an adjuvant can be injected into cancer patients in an attempt to induce a systemic immune response (b). The antigen presenting cell (APC) presents the antigen to T cell ((c) and (d)), thereby the T cell is activated (e) which results in the destruction of the cancer cell (f).

Active immunization seems to be one of the promising strategies to treat cancer though many approaches based on the employment of immune cells or immune molecules have been studied [57, 58]. In the last decade, this idea of vaccinations against cancer has transformed into clinical studies aiming to optimally deliver vaccines based on defined antigens to induce anticancer immunity. This method of treating cancerous cells relies on vaccines consisting of peptides derived from the protein sequence of candidate tumor-associated or specific antigens [57]. Tumor cells express antigens known as tumor-associated antigens (TAAs) that can be recognized by the host's immune system (T cells). Many TAAs have already been identified and molecularly characterized [59, 60]. These TAAs can be injected into cancer patients in an attempt to induce a systemic immune response that may result in the destruction of the cancer growing in different body tissues. This procedure is defined as active immunotherapy or vaccination as the host's immune system is either activated de novo or restimulated to mount an effective, tumor-specific immune reaction that may ultimately lead to tumor regression (Figure 3). Any protein/peptide produced in a tumor cell that has an abnormal structure due to mutation can act as a tumor antigen. Such abnormal proteins are produced due to mutation of the concerned gene. Various clinical studies focus on the therapeutic potential of active immunization or vaccination with TAA peptides in patients with metastatic cancer [61–63]. 

Most known TAAs are CTL (cytotoxic T lymphocyte also known as CD8+ T-cells or killer T cell) epitopes [64]. Peptide antigens are usually 8–10 amino acids long with 2-3 primary anchor residues that interact with MHC class 1-molecules and 2-3 residues which bind to T-cell receptor [64, 65]. CTLs directed against peptides presented by MHC class 1 molecules constitute powerful effectors of the immune system against tumor cells. The T-cell antigen receptor (TCR) on T cells recognizes the complex of a small peptide located in the antigen-binding groove of an MHC molecule [66]. MHC molecules (also called human leukocyte antigens (HLAs) in humans) are subdivided into class I molecules, which are found on all nucleated cells and class II molecules, which are found on specialized antigen-presenting cells (APCs) such as dendritic cells, macrophages, B cells, and selected activated endothelial or epithelial cells. CD4+ T cells recognize antigens bound to MHC class II molecules, and, as noted, class II molecules are expressed on APCs that possess the capability of antigen capture through phagocytosis or binding to surface antibody [67, 68]. 

Several of the peptide vaccines have undergone phase I and II clinical trials and have shown promising results in immunological as well as clinical responses. The notable peptide vaccines that have undergone phase I/II/III clinical trials include HER-2/neu immunodominant peptide (lung, breast, or ovarian cancer) [69–71], Mucin-1 (MUC-1, Stimuvax), peptide (breast or colon cancer) [72, 73], Carcinoembryonic antigen (colorectal, gastric, breast, pancreatic and non-small-cell lung cancers) [74, 75], Prostate-specific membrane antigen (prostate cancer) [76–78], HPV-16 E7 peptide (cervical cancer) [79], Ras oncoprotein peptide (colorectal and pancreatic carcinomas) [80–82], and Melanoma antigens (Melanoma) [62, 68, 83–85]. Another vaccine known as GV-1001 is under development, which is an injectable formulation of a promiscuous MHC class II peptide derived from the telomerase reverse transcriptase catalytic subunit (hTERT). GV-1001 is currently undergoing phase II clinical trials for liver cancer and NSCLC (non-small-cell lung cancer) as well as a phase III trial for pancreatic cancer [86].

The peptide vaccines are relatively less expensive, easy to manufacture and manipulate, are of defined structure, and being synthetic in nature do not have a problem of batch-to-batch variation. The major disadvantage of the peptide vaccines is their weak immunogenicity. Several strategies such as epitope enhancement, use of various T-cell epitopes, adjuvants, incorporation of costimulatory molecules, *ex vivo* loading into antigen presenting cells are being explored to enhance the immunogenicity and efficacy of the peptide vaccines [73–86].

## 5. Peptide as Cytotoxic Drug Carrier

Several peptide receptors are known which can be used as potential drug targets in cancer therapy [53–56, 87, 88]. The role of somatostatin receptors has already been discussed in the previous section for peptide receptor radionuclide therapy (PRRT). Similarly, a peptide can be conjugated to a cytotoxic drug to deliver it to a cancer cell expressing the corresponding peptide receptor. Such peptides are known as cell targeting peptides as they can specifically target a cell expressing its receptor. Cytotoxic compounds linked to analogs of hormonal peptides like LHRH, bombesin, and somatostatin can be targeted to certain tumors possessing receptors for those peptides and therefore are more selective for killing cancer cells [89, 90]. For example, a potential drug candidate, AEZS-108, couples a peptide LHRH with the chemotherapeutic agent doxorubicin to directly target cells that express LH-RH receptors, specifically prostate cancer cells [91, 92]. A list of different peptide receptors, their subtypes, tumors in which these receptors are expressed, and some of the targeting agents used are depicted in Table 2 [93]. Most of the studies so far are in the area of radionuclide therapy and imaging though a few studies examined the transport of cytotoxic drugs such as AN-201 and doxorubicin [94]. Nevertheless, these receptors provide good platform for the cell-specific delivery of chemotherapeutic agents. 

Apart from peptides that can selectively bind to the previous peptide receptors, many other peptides which are relevant to cancer treatment were discovered in recent years. These peptides obtained by *in vivo* phage display technology are known as homing peptides as they home very specifically to various normal organs or diseased tissues [95–97]. The RGD (Arg-Gly-Asp) and NGR (Asn-Gly-Arg) peptides represent the first generation of homing peptides [95]. Tumor homing of the RGD and NGR peptides appears to be independent of the tumor type, demonstrating that the receptors for these peptides are upregulated during angiogenesis. The RGD motif was originally discovered in peptides that bind to different integrins. The RGD peptide was shown to home to malignant melanoma, breast carcinoma xenografts, and rheumatoid arthritis model indicating that they recognize angiogenic vessels in general. The RGD peptides have high affinity towards the *α*_*v*_ integrin receptors in the angiogenic vasculature. The NGR motif was identified in an *in vivo* screen on human breast carcinoma xenografts [96]. It was originally identified as a cell adhesion motif, and it homes selectively to tumor blood vessels and other types of angiogenic vessels. The receptor for the NGR peptide is a peptidase, aminopeptidase N (APN), expression of which is upregulated in the angiogenic blood vessels. Several other peptides such as TAASGVRSMH and LTLRWVGLMS (chondroitin sulfate proteoglycan NG2 receptor) and F3 peptide (31 amino acid peptide that binds to cell surface-expressed nucleolin receptor) were identified thereafter [98, 99]. Recently, homing peptides with cell-penetrating ability were discovered [99–101]. Cell-penetrating peptides (CPPs) are small peptides, generally less than 30 amino acids long, that internalize very efficiently into all cells they come into contact with [102]. These internalizing homing peptides are similar to the classic cell-penetrating peptides, such as the transcription-transactivating (Tat) protein of HIV-1, and penetrating with an important exception: the internalization of the homing peptides is cell-type-specific. Both the F3 and LyP-1 (CGNKRTRGC) peptides are cell-type-specific CPPs [99–101]. They are able to internalize tumor cells and blood (F3) or lymphatic (LyP-1) endothelial cells in the tumors they home to.

Homing peptides have been successfully used as delivery vehicles to target imaging agents, drug molecules, oligonucleotides, liposomes, and inorganic nanoparticles to tumors and other tissues [97, 103, 104]. One drug that has been delivered using RGD and NGR peptides is the tumor necrosis factor-*α* (TNF-*α*) that has potent antitumor activity [98, 105]. The clinical use of TNF-*α* itself as an anticancer drug is limited to local treatments due to its dose-limiting systemic toxicity. RGD and NRG peptide-targeted TNF-*α* treatment decreased tumor growth with smaller doses than free TNF-*α*. The antitumor activity of NGR-TNF-*α* was also studied in combination with various chemotherapeutic drugs: doxorubicin and melphalan as well as cisplatin, paclitaxel, and gemcitabine and compared to the efficacy of the chemotherapeutic drugs alone in various murine tumor models [105]. The results showed that targeted delivery of low doses of NGR-TNF-*α* to tumor vasculature increased the efficacy of various drugs acting via different mechanisms. Moreover, transgenic mice with androgen-independent prostate carcinoma (TRAMP-C1) were treated with repeated cycles of doxorubicin, administered either alone or following NGR-TNF-*α* administration. Pretreatment with NGR-TNF-*α* significantly expanded the therapeutic index of doxorubicin and significantly delayed tumor growth without increasing drug-related toxicity. The RGD homing peptide has also been conjugated to doxorubicin [97, 106]. This treatment inhibited tumor growth and prolonged the lifespan of tumor-bearing animals. Again the doxorubicin-RGD conjugate was less toxic than doxorubicin alone or doxorubicin conjugated to a control peptide. Conjugation of IL-12 (Interleukin-12) to the CDCRGDCFC (RGD-4C) peptide, a specific ligand for *α*_*v*_ *β*3 integrin, targets IL-12 directly to tumor neovasculature [107]. This fusion protein stimulated interferon-*γ* production *in vitro* and *in vivo*, suggesting biological activity consistent with IL-12. Localization of IL-12 to the angiogenic vasculature significantly enhanced the antiangiogenic effect in corneal angiogenesis assay, augmented antitumor activity in a neuroblastoma model, and decreased toxicity of the IL-12.

A number of clinical trials based on RGD and NGR targeted drug delivery are currently ongoing or recruiting patients [108–110]. For example, a phase Ib study was conducted to verify the safety of NGR peptide-targeted hTNF in combination with doxorubicin in treatment of refractory/resistant solid tumors [108]. Fifteen patients received various doses of a combination of NGR-targeted hTNF and doxorubicin intravenously. One partial response (7%) and ten stable diseases (66%) lasting for a median duration of 5.6 months were observed. These results prompted plans for the phase II development (http://clinicaltrials.gov/). In addition, a phase III trial on newly diagnosed glioblastoma has been started [97]. A recently identified peptide called iRGD is able to specifically recognize and penetrate cancerous tumors but not normal tissues [111]. Chlorotoxin (a 36 amino acid peptide derived from scorpion venom) binds preferentially to glioma cells compared with nonneoplastic cells or normal brain has allowed the development of new methods for the treatment and diagnosis of cancer [112].

## 6. Anticancer Peptides

Direct use of peptide as a therapeutic agent to treat cancer is gaining momentum in the recent years. Anticancer activity of different peptides is attributed to a variety of mechanisms that restrict tumor growth. The mechanism involves the inhibition of angiogenesis, protein-protein interactions, enzymes, proteins, signal transduction pathways, or gene expression [113–120]. Another category of anti-cancer peptides is peptide antagonists which can preferentially bind to a known receptor [121, 122]. Moreover “pro-apoptotic” peptides mediate significant induction of apoptosis (programmed cell death) in tumors [123–125]. 

Angiogenesis involves the migration, growth, and differentiation of endothelial cells, which line the inside wall of blood vessels. There is a tremendous effort to discover angiogenesis inhibitors, based on peptides as the safest and least toxic therapy for diseases associated with abnormal angiogenesis [113]. Angiogenesis requires the binding of signaling molecules, such as vascular endothelial growth factor (VEGF), to receptors on the surface of normal endothelial cells. When VEGF and other endothelial growth factors bind to their receptors on endothelial cells, signals within these cells are initiated that promote the growth and survival of new blood vessels. Angiogenesis inhibitors interfere with various steps in this process. A number of ongoing clinical trials in this area focus on peptides derived from extracellular matrix proteins, growth factors and growth factor receptors, coagulation cascade proteins, chemokines, and type I Thrombospondin domain containing proteins and serpins [113, 114]. Recently it was found that angiotensin-(1–7) can stop lung cancer tumor growth in mice by inhibiting blood vessel formation [126]. The antiangiogenic agent cilengitide (Merck) is a derivative of the RGD peptide [127–130]. It is the inner salt of a cyclized RGD pentapeptide (cyclo-[Arg-Gly-Asp-DPhe-(NMeVal)]) that is selective for *α*v integrins, which are important in angiogenesis. It is currently under phase II trial for the treatment of glioblastoma and refractory brain tumors in children. Another peptide, ATN-161 (Ac-PHSCN-NH2), is in early phase II trials for cancer [120]. It binds to and inhibits integrins involved in tumor progression (a5ß1, avß3, and avß5), while not inhibiting adhesion as it is not based on the RGD sequence [131]. A dipeptide, L-glutamine L-tryptophan (IM862) that is made normally in the thymus gland, has shown antiangiogenic properties. Though it was shown recently to be ineffective against AIDS-Kaposi's sarcoma in a phase III trial, it still holds promise for other forms of cancer [132, 133].

BN/GRP (bombesin/gastrin-releasing peptide) peptides were shown to bind selectively to the G-protein-coupled receptors on the cell surface, stimulating the growth of various malignancies in murine and human cancer models [134, 135]. Thus, it has been proposed that the secretion of BN/GRP by neuroendocrine cells might be responsible for the development and progression of prostate cancer to androgen independence. GRP is widely distributed in lung and gastrointestinal tracts. It is produced in small cell lung cancer (SCLC), breast, prostatic, and pancreatic cancer and functions as a growth factor. The involvement of bombesin-like peptides in the pathogenesis of a wide range of human tumors, their function as autocrine/paracrine tumoural growth factors, and the high incidence of BN/GRP receptors in various human cancers prompted the design and synthesis of BN/GRP receptor (GRPR) antagonists such as RC-3095, RC-3940-II, and RC-3950 [136–138]. Recently, many researchers are focusing on the development of GHRH (growth hormone releasing hormone—a hypothalamic polypeptide) antagonists as potential anti-cancer therapeutics since the GHRH is produced by various human tumors, including prostate cancer, and seems to exert an autocrine/paracrine stimulatory effect on tumors [139, 140]. 

Recently, scientists have designed peptides to target the protein-protein interface of a key enzyme in DNA synthesis crucial for cancer growth [141]. The peptides act by a novel inhibitory mechanism and curb cancer cell growth in drug-resistant ovarian cancer cells. These octapeptides specifically target the protein-protein interface of thymidylate synthase. Thymidylate synthase is composed of two identical polypeptide chains; that is, it is a homodimer. The peptides stabilize the inactive form of the enzyme, show a novel mechanism of inhibition for homodimeric enzymes, and inhibit cell growth in drug sensitive and resistant cancer cell lines [141].

Cisplatin, cisplatinum, or *cis*-diamminedichloroplatinum(II) is the first member of a class of platinum-containing anti-cancer drugs. These platinum complexes react *in vivo*, binding to and causing crosslinking of DNA, which ultimately triggers apoptosis (programmed cell death). Recently, Pt (IV)-peptide conjugates were found to be good inhibitors of cellular proliferation when compared to a nontargeting platinum(IV) parent compound, showing that its relatively low cytotoxicity is greatly improved by addition of the peptides [142]. (KLAKLAK)2 is an antimicrobial apoptosis-inducing peptide that upon internalization causes mitochondrial swelling and disruption of the mitochondrial membrane leading to apoptosis [123, 124, 143]. The RGD-(KLAKLAK)2 and NGR-(KLAKLAK)2 were especially toxic to angiogenic endothelial cells leading to reduced tumor growth and metastases as well as prolonged survival. LyP-1 is unique among the tumor homing peptides since it has cytotoxic activity on its own [144]. When injected into the tail vein of mice with MDA-MB-435 breast cancer xenografts, LyP-1 peptide by itself inhibited tumor growth and reduced the number of lymphatic vessels, thus demonstrating a cytotoxic activity of the peptide.

## 7. Other Anticancer Drugs Closely Related to Peptides

Bortezomib is the first therapeutic proteasome inhibitor to be tested in humans [21, 145, 146]. It is approved in the USA for treating relapsed multiple myeloma and mantle cell lymphoma (2003). In multiple myeloma, complete clinical responses have been obtained in patients with otherwise refractory or rapidly advancing disease. The drug is an N-protected dipeptide and can be written as Pyz-Phe-boroLeu, which stands for pyrazinoic acid, phenylalanine, and leucine with a boronic acid instead of a carboxylic acid. Mifamurtide (Mepact) is a drug against osteosarcoma, which is lethal in about a third of cases [147]. The drug was approved in Europe in March 2009 and is not currently approved in the USA. Mifamurtide is a fully synthetic derivative of muramyl dipeptide (MDP) the smallest naturally occurring immune stimulatory component of cell walls from *Mycobacterium* species. The side chains of the molecule give it a longer elimination half-life than the natural substance. Being a phospholipid, it accumulates in the lipid bilayer of the liposomes in the infusion. It recognizes muramyl dipeptide and simulates a bacterial infection by binding to NOD2 (NOD2 is a pattern recognition receptor which is found in several kinds of white blood cells, mainly monocytes and macrophages) activating white cells [148]. This results in an increased production of TNF-*α*, interleukin 1, interleukin 6, interleukin 8, interleukin 12, and other cytokines, as well as ICAM-1. The activated white cells attack cancer cells, but not other cells. Brentuximab Vedotin, an antibody drug conjugate (ADS) approved in 2011, is a chimeric monoclonal antibody, brentuximab (which targets the cell-membrane protein CD30) linked to three to five units of the antimitotic agent monomethyl auristatin E. The linker here is a valine-citrulline dipeptide which is cleaved by cathepsin once the conjugate has entered a tumor cell. The antimitotic agent monomethyl auristatin E can be considered as a peptidomimetic [149, 150]. 

## 8. Conclusion

In conclusion, peptides are poised to make a huge impact in near future in the area of cancer treatment and diagnosis. Targeted chemotherapy and drug delivery techniques are emerging as an excellent tool in minimizing problems with the conventional chemotherapy. Along with different peptide-based cancer therapeutics already available for patients, a number of peptide-based therapies such as cancer vaccines, tumor targeting with cytotoxic drugs and radioisotopes, and anti-angiogenic peptides are currently on clinical trials and are expected to yield positive results. Stimuvax (palmitoylated peptide vaccine against nonsmall lung cancer, Merck), Primovax (peptide cancer vaccine, Pharmexa), Melanotan (precancerous actinic keratosis, Clinuvel), and Cilengitide (Glioblastoma, Merck) are some examples of potential peptides in late clinical trials. Due to the tremendous advancement in the large scale synthesis of peptides it will be possible to make peptide-based anti-cancer drugs more affordable to patients. In recent years combination therapy is emerging as an important strategy to fight cancer as just one method may not be efficient enough to cure the disease completely or prevent recurrence [151]. In the hope of achieving synergistic effects, combinations of antiangiogenesis with traditional chemotherapy are currently being pursued in clinical trials [151–155]. For example, cilengitide was used in a phase I/IIa combination trial, which combined cilengitide with radiotherapy and temozolomide for newly diagnosed Glioblastoma patients resulting in better overall survival (OS) rates [152]. ATN-161 enhances the activity of radiation and chemotherapy and is progressing to a phase II trial for head and neck cancer [153]. Encouraging data are emerging that strongly support the notion that combining immunotherapy with conventional therapies, for example, radiation and chemotherapy, may improve efficacy [154] in cancer treatment and management. 

## Figures and Tables

**Table 1:** LHRH agonists and new generation antagonists available in the market.

Peptide	Sequence comparison	Indications
Agonists		
Buserelin	Pyr-His-Trp-Ser-Tyr-D-Ser(OtBu)-Leu-Arg-Pro-NHEt	Prostate cancer
Gonadorelin	Pyr-His-Trp-Ser-Tyr-Gly-Leu-Arg-Pro-Gly-NH_2_	Cystic ovarian disease, agent for evaluating hypothalamic-pituitary gonadotropic function
Goserelin	Pyr-His-Trp-Ser-Tyr-D-Ser(OtBu)-Leu-Arg-Pro-AzGly-NH_2_	Prostate cancer; breast cancer
Histrelin	Pyr-His-Trp-Ser-Tyr-D-His(N-benzyl)-Leu-Arg-Pro-NHEt	Prostate cancer; breast cancer
Leuprolide	Pyr-His-Trp-Ser-Tyr-D-Leu-Leu-Arg-Pro-NHEt	Prostate cancer; breast cancer
Nafarelin	Pyr-His-Trp-Ser-Tyr-2Nal-Leu-Arg-Pro-Gly-NH_2_	Treat symptoms of endometriosis, central precocious puberty
Triptorelin	Pyr-His-Trp-Ser-Tyr-D-Trp-Leu-Arg-Pro-Gly-NH_2_	Prostate cancer; breast cancer

Antagonists		
Abarelix	Ac-D-2Nal-D-4-chloroPhe-D-3-(3′-pyridyl) Ala-Ser-(N-Me)Tyr-D-Asn-Leu-isopropylLys-Pro-DAla-NH_2_	Prostate cancer
Cetrorelix	Ac-D-2Nal-D-4-chloroPhe-D-3-(3′-pyridyl) Ala-Ser-Tyr-D-Cit-Leu-Arg-Pro-D-Ala-NH_2_	Prostate cancer; breast cancer
Degarelix	Ac-D-2Nal-D-4-chloroPhe-D-3-(3′-pyridyl) Ala-Ser-4-aminoPhe(L-hydroorotyl)-D-4-aminoPhe(carbamoyl)-Leu-isopropylLys-Pro-D-Ala-NH_2_	Prostate cancer
Ganirelix	Ac-D-2Nal-D-4-chloroPhe-D-3-(3′-pyridyl) Ala-Ser-Tyr-D-(N9, N10-diethyl)-homoArg-Leu-(N9, N10-diethyl)-homoArg-Pro-D-Ala-NH_2_	Fertility treatment

**Table 2:** Peptide receptors which have potential in cancer therapy.

Peptide receptors	Receptor subtypes	Expressing tumor type	Targeting agents
Somatostatin	sst1, sst2, sst3, sst4, and sst5	GH-producing pituitary adenoma, paraganglioma, nonfunctioning pituitary adenoma, pheochromocytomas	Radioisotopes, AN-201 (a potent cytotoxic radical 2-pyrrolinodoxorubicin), doxorubicin
Pituitary adenylate cyclase activating peptide (PACAP)	PAC1	Pheochromocytomas and paragangliomas	Radioisotopes, doxorubicin
Vasoactive intestinal peptide (VIP/PACAP)	VPAC1, VPAC2	Cancers of lung stomach, colon, rectum, breast, prostate, pancreatic ducts, liver, and urinary bladder	Radioisotopes, camptothecin
Cholecystokinin (CCK)	CCK1 (formerly CCK-A) and CCK2	Small cell lung cancers, medullary thyroid carcinomas, astrocytomas, and ovarian cancers	Radioisotopes, cisplatin
Bombesin/gastrin-releasing peptide (GRP)	BB1, GRP receptor subtype (BB2), the BB3 and BB4	Renal cell, breast, and prostate carcinomas	Doxorubicin, 2-pyrrolinodoxorubicin
Neurotensin	NTR1, NTR2, NTR3	Small cell lung cancer, neuroblastoma, pancreatic and colonic cancer	Radioisotopes
Substance P	NK1 receptor	Glial tumors	Radioisotopes
Neuropeptide Y	Y1–Y6	Breast carcinomas	Radioisotopes
